# Anti-Obese Effect of Glucosamine and Chitosan Oligosaccharide in High-Fat Diet-Induced Obese Rats

**DOI:** 10.3390/md13052732

**Published:** 2015-04-30

**Authors:** Lanlan Huang, Jian Chen, Peiqiu Cao, Haitao Pan, Chen Ding, Tiancun Xiao, Pengfei Zhang, Jiao Guo, Zhengquan Su

**Affiliations:** 1 Key Research Center of Liver Regulation for Hyperlipidemia SATCM/Class III Laboratory of Metabolism SATCM, Guangdong TCM Key Laboratory for Metabolic Diseases, Guangdong Pharmaceutical University, Guangzhou 510006, China; E-Mails: lanlanhuangle@163.com (L.H.); yx07228chenjian@126.com (J.C.); cpq_520@126.com (P.C.); pangel7835001@163.com (H.P.); 407874729@qq.com (C.D.); 2 Inorganic Chemistry Laboratory, Oxford University, South Parks Road, OX1 3QR Oxford, UK; E-Mail: xiao.tiancun@chem.ox.ac.uk; 3 Guangzhou Boxabio Technology Ltd., Guangzhou Hi-Tech Development Zone, Guangzhou 510663, China; E-Mail: Pengfei.Zhang@boxenergytech.com

**Keywords:** GLC, COS1, COS2, PPARγ, LXRα, anti-obesity

## Abstract

Objective: This study is to evaluate the anti-obese effects of glucosamine (GLC) and chitosan oligosaccharide (COS) on high-fat diet-induced obese rats. Methods: The rats were randomly divided into twelve groups: a normal diet group (NF), a high-fat diet group (HF), Orlistat group, GLC high-, middle-, and low-dose groups (GLC-H, GLC-M, GLC-L), COS1 (COS, number-average molecular weight ≤1000) high-, middle-, and low-dose groups (COS1-H, COS1-M, COS1-L), and COS2 (COS, number-average molecular weight ≤3000) high-, middle-, and low-dose groups (COS2-H, COS2-M, COS2-L). All groups received oral treatment by gavage once daily for a period of six weeks. Results: Rats fed with COS1 gained the least weight among all the groups (*P* < 0.01), and these rats lost more weight than those treated with Orlistat. In addition to the COS2-H and Orlistat groups, the serum total cholesterol (CHO) and low-density lipoprotein cholesterol (LDL-C) levels were significantly reduced in all treatment groups compared to the HF group (*P* < 0.01). The various doses of GLC, COS1 and COS2 reduced the expression levels of PPARγ and LXRα mRNA in the white adipose tissue. Conclusions: The results above demonstrated that GLC, COS1, and COS2 improved dyslipidemia and prevented body weight gains by inhibiting the adipocyte differentiation in obese rats induced by a high-fat diet. Thus, these agents may potentially be used to treat obesity.

## 1. Introduction

Obesity is a chronic metabolic disease caused by an energy imbalance that results in excess body fat accumulation. In 1948, the World Health Organization (WHO) noted that obesity was a disease state and classified it as an official disease of endocrinology in 1997 [1]. Due to improvements in living standards and changes in life style, including eating habits, the number of obese individuals has rapidly increased worldwide. In America, approximately 66% adults are overweight or obese, and the incidence rate of obesity is 32% [2]. In China, 11.4% and 10.1% of men and women were obese in 2009, respectively, and the male and female abdominal obesity ratio has also increased to 27.8% and 45.9%, respectively [3]. Many studies have demonstrated that obesity may be due to genetics, diet, environment, living traditions and other factors [4], and it serves as a predisposing factor to non-alcoholic fatty liver disease (NAFLD), type II diabetes, atherosclerosis, cancer, sleep disorders, osteoarthritis and other diseases [5,6,7]. Therefore, safe and effective weight-loss drugs are urgently needed to treat obesity. The therapies reported for the treatment of obesity are abundant and include diet, drug, exercises, and surgical and behavioral therapies. As the main treatment for adiposity, drug therapy features high compliance and is more convenient and safer than surgical therapy. However, some drugs have been withdrawn from the market due to unexpected side effects, such as Pondimin, Redux, Acomplia, and Meridia. Lorcaserin and Belviq were approved in 2012, and only Orlistat is used as an over the counter in weight loss aid. Many trials have been recently conducted to find and develop new anti-obese drugs based on Chinese traditional medicine or natural compounds because of their high activity, novel structure and potentially less severe side effects.

Chitosan oligosaccharide (COS) is a homopolymer or different polymer formed by the β-1,4 glycosidic linkage of glucosamine and *N*-acetyl glucosamine, a derivative of chitosan (CTS) with 2–10 degrees of polymerization [8]. The human intestinal absorption rate of COS is close to 100% due to its good water solubility, and COS is biocompatible and non-toxic to humans. In recent years, Chinese and international studies revealed that COS was an ideal bioactive substance and an effective neuroprotection, anti-cancer, antibacterial, anti-inflammatory, hypoglycemic, antioxidant, and liver protection agent [9,10,11,12,13,14,15]. In addition, COS is also used in the field of food and nutrition to reduce lipid levels, serving as an effective lipid-lowering dietary supplement [16].

As the ultimate degradation products of CTS, glucosamine (GLC) is abundant in the tendons, cartilage and ligaments of humans and animals that can synthesize proteoglycan and collagen, and it protects cartilage tissue. A great number of studies have shown that GLC positively affects osteoarthritis [17,18] and can improve the symptoms of inflammation. Further research has also found that GLC effectively improved liver injury induced by carbon tetrachloride [19], activates macrophages to enhance immunity [20], acts as an antiseptic and antibacterial [21], induces autophagy to prevent neurodegenerative diseases and scavenges free radicals in mice [22,23].

Digital gene expression tag profiling (DGE) is an accurate, comprehensive and rapid sequencing method used to detect differences in gene expression in specific tissues with characterization in testing samples and high repeatability [24]. Gene ontology (GO) is an internationally accepted means of gene function analysis that describes the molecular function of the gene (Molecular Function), including the in cell location (Cellular Component) and biological processes involved (Biological Process) [25]. Pathway significant functional enrichment analysis differs from in that it is based on the KEGG Pathway as a unit and uses a hypergeometric test method to determine the metabolic pathways in which genes are differentially expressed and signal transduction pathway genes are involved [26,27].

We have studied the anti-obese effects of various CTS in our previous studies, including water-soluble CTS [28,29], CTS microspheres, and chitosan-capsaicin microspheres [30]. However, CTS is poorly soluble and does not encapsulate drugs well. So far, the anti-obese effect of COS is being extensively and intensively investigated. Studies have shown that COS significantly decreased lipid accumulation, a marker of adipogenesis, in a dose dependent manner [31]. The low molecular mass COS (1–3 kDa) were more effective in inhibiting adipocyte differentiation in 3T3-L1 cells [32,33]. COS treatment notably decreased the expression of peroxisome proliferator-activated receptor γ (PPARγ), a key adipogenic transcription factor. COS also significantly down-regulated adipogenic marker proteins, such as leptin, adiponectin, and resistin [31]. However, the anti-obese activity of COS has not been compared with other compounds. As a drug for osteoarthritis, GLC is the ultimate degradation product of CTS. Could GLC and low molecular weight COS be potential and natural anti-obese drugs that are superior to the chemical drugs already in use on the market?

In this study, we have investigated into the anti-obesity properties of GLC, COS1 and COS2 in a nutrition-induced animal model of obesity. We assessed the effect of COS molecular weight on its anti-obese activity and simultaneously explored the anti-obese activity of GLC. DGE was used to identify potential changes in gene sequencing caused by GLC and COS1. GO and Pathway significant functional enrichment analysis were carried to identify the potential pathways activated by GLC and COS1 during weight loss, which constitutes the foundation to the future study. Furthermore, the LXRα gene is a known direct target of PPARγ, and these nuclear receptors cooperate in the regulation of lipid metabolism [34]. Therefore, the expressions of rat epididymal adipose tissues PPARγ and LXRα mRNA were measured using real-time quantitative PCR to explore the pharmacological mechanisms of GLC, COS1 and COS2.

## 2. Results and Discussion

### 2.1. Food Intake

**Figure 1:**
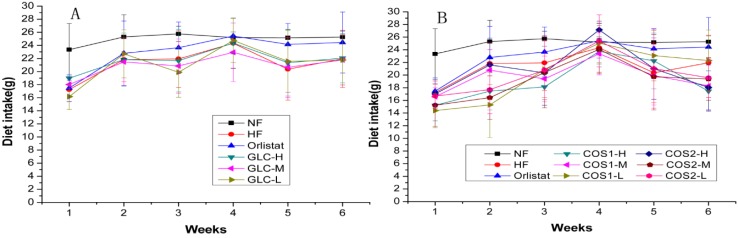
The effect of GLC (**A**), COS1 and COS2 (**B**) to rats’ weekly diet intake during administration (*n* = 10, means ± SD). Food intake of rats gradually increased for four weeks and then declined during the following two weeks. In Figure 1B, the food intake of rats was decreased in the COS1-H, COS1-M, COS1-L, COS2-M and COS2-L groups when compared with Orlistat. Abbreviations: GLC, glucosamine; COS, chitosan oligosaccharide; COS1, number-average molecular weight ≤1000; COS2, number-average molecular weight ≤3000; NF, normal diet group; HF, high-fat diet group; GLC-H, GLC-M, GLC-L, GLC high-, middle-, and low-dose groups; COS1-H, COS1-M, COS1-L, COS1 high-, middle-, and low-dose groups; COS2-H, COS2-M, COS2-L, COS2 high-, middle-, and low-dose groups.

The food intake of the rats during the administration period is shown in Table 1 and Figure 1. Compared with HF, the test samples did not affect the appetite of rats, except for the COS1-H group. However, compared with Orlistat, the food intake of rats was decreased in the COS1-H, COS1-M, COS1-L, COS2-M and COS2-L groups, but not in the GLC group. Figure 1 shows the effect of GLC, COS1 and COS2 on the weekly dietary intake of rats during the administration period. Food intake of rats slowly increased for four weeks and then declined during the following two weeks. The results in Table 1 show that GLC and COS2 did not affect rats, and weight loss was likely not due to a reduction in appetite.

**Table 1:** The effect of each drug to the rats’ dietary intake during the administration period (*n* = 10, means ± SD).

Groups	Dietary Intake during Administration (g/day)
NF	25.07 ± 2.40 **
HF	21.27 ± 4.14
Orlistat	22.99 ± 4.29
GLC-H	21.70 ± 3.94
GLC-M	20.95 ± 3.77
GLC-L	21.14 ± 4.30
COS1-H	19.04 ± 4.05 *^##^
COS1-M	19.72 ± 3.84 ^##^
COS1-L	20.17 ± 5.12 ^#^
COS2-H	20.86 ± 4.75
COS2-M	19.21 ± 4.81 ^##^
COS2-L	20.19 ± 4.25 ^#^

Note: Compared with the HF group, * *P* < 0.05; ** *P* < 0.01; Compared with Orlistat, ^#^ *P* < 0.05, ^##^ *P* < 0.01.

### 2.2. Body Weight

**Figure 2:**
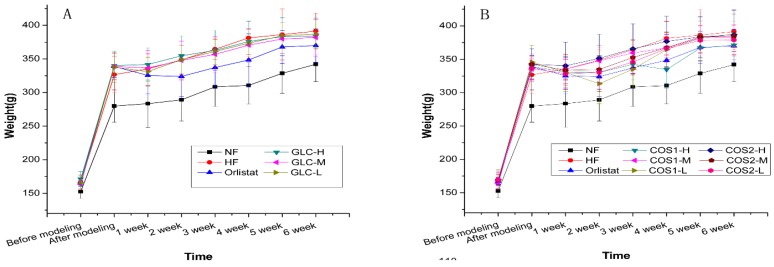
The effect of GLC (**A**), COS1 and COS2 (**B**) to rats’ weight (*n* = 10, means ± SD). After modeling, the weight of all rats increased, but NF group had lower gain, which significantly differed from those of the other groups. The treatment groups tended to gain weight slower than the HF group, which indicated that GLC, COS1 and COS2 could inhibit weight gain in rats.

The body weight of rats fed with a high-fat diet was 21.08% greater than that of the rats fed with a normal diet, indicating that the model was successfully established. Figure 2A,B show that the weight of the NF group significantly differed from those of the other groups before and after the establishment of the model. Drug treatment appeared to slow weight gain compared with the HF group. The weekly weight gain was similar for the COS1-H and Orlistat groups. The effect of GLC, COS1 and COS2 on weight gain in rats is demonstrated in Table 2. The rats in the treatment group gained significantly less weight than those in the HF group: GLC (*P* < 0.05), COS2 (*P* < 0.05), COS1-H (*P* < 0.01), and COS1-L (*P* < 0.05). This finding indicated that GLC, COS1 and COS2 could inhibit weight gain in rats, as shown in Figure 2 and Table 2. COS1-H was more effective in reducing body weight than Orlistat. COS1-H was more effective than COS2-H, and this effect was dose-dependent.

**Table 2:** The effect of drugs on weight gain in the rats (*n* = 10, means ± SD).

Groups	Weight before Modeling (g)	Weight before Administration (g)	Weight after Administration (g)	Weight Gain during Administration(g)
NF	152.78 ± 10.34 **^##^	279.92 ± 24.07 **^##^	342.34 ± 25.51 **^##^	62.42 ± 14.52 ^##^
HF	166.39 ± 11.49	337.01 ± 20.35	397.38 ± 18.00 ^#^	60.37 ± 26.39 ^##^
Orlistat	167.04 ± 5.84	335.29 ± 20.08	369.99 ± 24.74 *	34.70 ± 19.48 **
GLC-H	171.31 ± 11.07	340.18 ± 21.24	383.91 ± 27.70	43.74 ± 14.14 *
GLC-M	163.51 ± 5.65	338.81 ± 19.44	381.90 ± 27.57	43.09 ± 11.87 *
GLC-L	165.61 ± 10.22	337.77 ± 22.20	387.19 ± 21.32	49.42 ± 12.56 ^#^
COS1-H	168.27 ± 12.41	338.96 ± 13.79	371.10 ± 16.72 *	32.14 ± 14.60 **
COS1-M	163.64 ± 9.40	335.09 ± 15.22	382.11 ± 13.07	47.03 ± 13.70
COS1-L	170.31 ± 14.16	345.76 ± 24.98	387.59 ± 29.98	41.83 ± 8.93 *
COS2-H	168.96 ± 9.96	342.53 ± 23.31	386.59 ± 37.20	44.06 ± 20.46 *
COS2-M	169.16 ± 7.42	335.29 ± 20.16	384.10 ± 22.72	41.39 ± 14.28 *
COS2-L	169.64 ± 11.46	336.36 ± 19.61	378.87 ± 22.42	42.51 ± 10.31 *

Note: Compared with the HF group, * *P* < 0.05; ** *P* < 0.01; Compared with Orlistat, ^#^ *P* < 0.05; ^##^ *P* < 0.01.

### 2.3. Organ Index

The hearts and livers of rats were excised after drug treatment, and the organ index was calculated. The influences of GLC, COS1 and COS2 on the heart and liver fat deposition were observed, and the results are shown in Table 3. GLC, COS1 and COS2 minimally affected the cardiac index and significantly affected the liver index of rats compared to the HF group. Table 3 shows that the liver index of the HF group significantly differed from those of groups COS1-H (*P* < 0.05), COS1-M (*P* < 0.01), COS1-L (*P* < 0.05) and COS2-H (*P* < 0.05), and COS2-L (*P* < 0.01); the liver index of the GLC group showed less difference from that of the HF group. Thus, COS1 and COS2 may reduce the deposition of fat in the liver and are more effective than Orlistat.

**Table 3:** The effect of drugs on organ index in rats (*n* = 10, means ± SD).

Groups	Cardiac Index (%)	Liver Index (%)
NF	0.32 ± 0.02 ^#^	2.41 ± 0.19 *
HF	0.33 ± 0.03	2.59 ± 0.28
Orlistat	0.30 ± 0.03	2.44 ± 0.23
GLC-H	0.31 ± 0.02	2.75 ± 0.17 ^##^
GLC-M	0.32 ± 0.02	2.55 ± 0.15
GLC-L	0.33 ± 0.03 ^#^	2.53 ± 0.18
COS1-H	0.33 ± 0.02	2.39 ± 0.16 *
COS1-M	0.33 ± 0.03 ^#^	2.35 ± 0.23 **
COS1-L	0.32 ± 0.03	2.41 ± 0.10 *
COS2-H	0.32 ± 0.03	2.38 ± 0.18 *
COS2-M	0.30 ± 0.01	2.41 ± 0.16
COS2-L	0.31 ± 0.03	2.34 ± 0.12 **

Note: Compared with HF, * *P* < 0.05; ** *P* < 0.01; Compared with Orlistat, ^#^ *P* < 0.05; ^##^ *P* < 0.01.

### 2.4. Fat Pad and Body Fat Ratio

**Figure 3:**
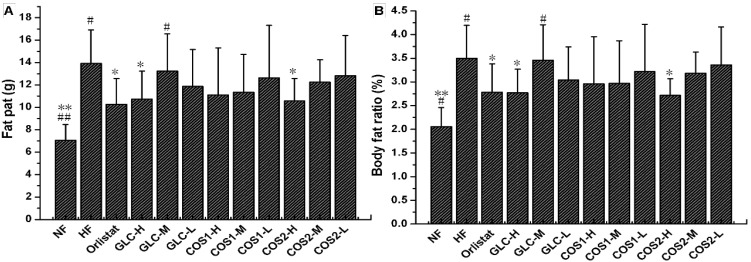
The effect of GLC, COS1 and COS2 on fat pat (**A**) and body fat (**B**) ratio in rats (*n* = 10, means ± SD). Orlistat, GLC-H, and COS2-H (*P* < 0.05) could reduce the body fat and body fat ratio in obese rats when compared with the HF group, which carried high fat. Besides, it appeared dose dependent in the COS2 group. Note: Compared with HF, * *P* < 0.05; ** *P* < 0.01; Compared with Orlistat, ^#^ *P* < 0.05; ^##^ *P* < 0.01.

After the drug treatment period, the perirenal fat and epididymal fat samples were taken to calculate the wet weight of the fat pad and body fat ratio. The wet weight of the fat pad in the HF group exceeded that of rats in the NF group, indicating that the fat content of rats in the HF group was high (Figure 3). Compared with the HF group, Orlistat, GLC-H, and COS2-H (*P* < 0.05) could reduce the body fat and body fat ratio in obese rats. COS1 could also reduce rat body fat, but this reduction was not significant.

### 2.5. Serum Lipid Determination

**Figure 4:**
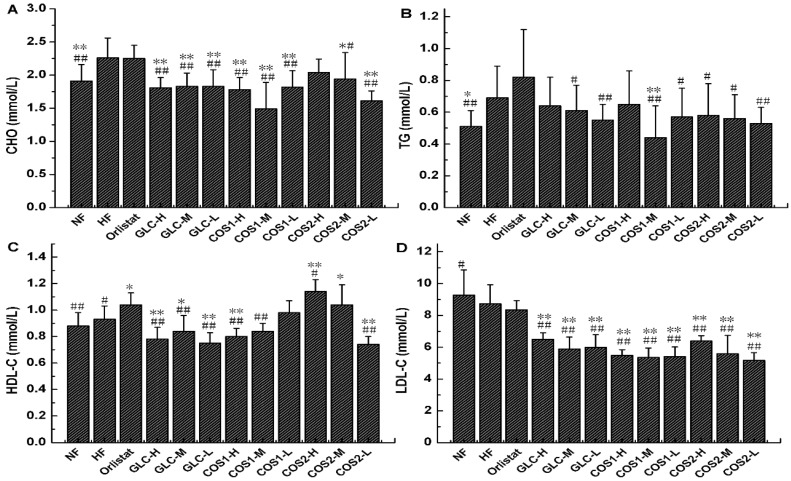
The effect of GLC, COS1 and COS2 on serum CHO, TG, HDL-C and LDL-C in rats (*n* = 10, means ± SD). (**A**) GLC, COS1 and COS2 could reduce the CHO in the serum, which were better than Orlistat; (**B**) Compared with the HF group, the TG in the serum was decreased in GLC, COS1 and COS2; (**C**) COS2-H and COS2-M significantly increased the HDL-C in the serum, and it appeared dose dependent in the COS1 group; (**D**) GLC, COS1 and COS2 reduced the LDL-C in the serum more effectively than Orlistat. Note: Compared with HF, * *P* < 0.05; ** *P* < 0.01; Compared with Orlistat, ^#^ *P* < 0.05; ^##^ *P* < 0.01.

The lipid levels in the serum were detected using an automatic biochemical analyzer, and the results are shown in Figure 4. GLC, COS1 (*P* < 0.01), COS2-M and COS2-L significantly reduced (*P* < 0.01) the CHO in the serum compared with the HF group, and this effect was better than that elicited by Orlistat. COS1-M (*P* < 0.01) significantly reduced the TG in the serum. COS2-H (*P* < 0.01) and COS2-M (*P* < 0.05) significantly increased the HDL-C in the serum. GLC (*P* < 0.01), COS1 (*P* < 0.01) and COS2 (*P* < 0.01) reduced the LDL-C in the serum more effectively than Orlistat. The results in Figure 4 show that GLC, COS1 and COS2 reduced the CHO and LDL-C levels in the serum better than Orlistat; COS1-H and COS1-M were more effective than COS2-H and COS2-M.

### 2.6. Histological Analysis

**Figure 5:**
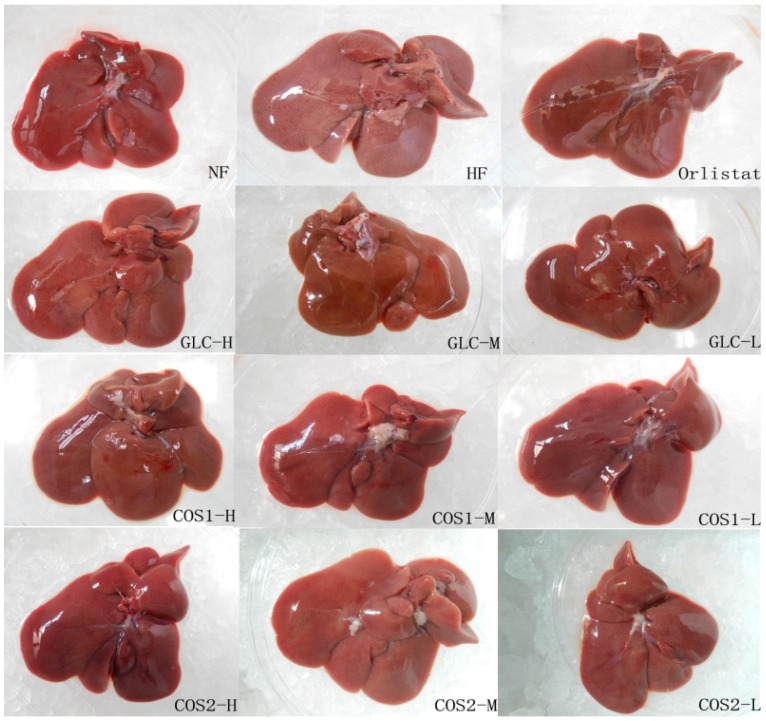
The effect of GLC, COS1 and COS2 on liver morphologies in the rats. The livers of rats in the NF group were small, bright red, smooth and had sharp edges. In contrast, those in the HF group were light yellow, swelling, thick edge and had plenty of visible fat granules. However, this state of fatty liver was relieved in treatment groups, implying that GLC, COS1 and COS2 could reduce fat deposition in the liver, and protect the liver.

The liver morphology (Figure 5) showed that the livers of rats in the Orlistat, GLC, COS1 and COS2 groups were small, contained few fat particles, were bright red in color, and featured with sharp edges and smooth section, similar to those in the NF group. GLC, COS1 and COS2 were shown to accelerate the metabolism of fat in the liver, reduce fat deposition, and protect the liver.

**Figure 6:**
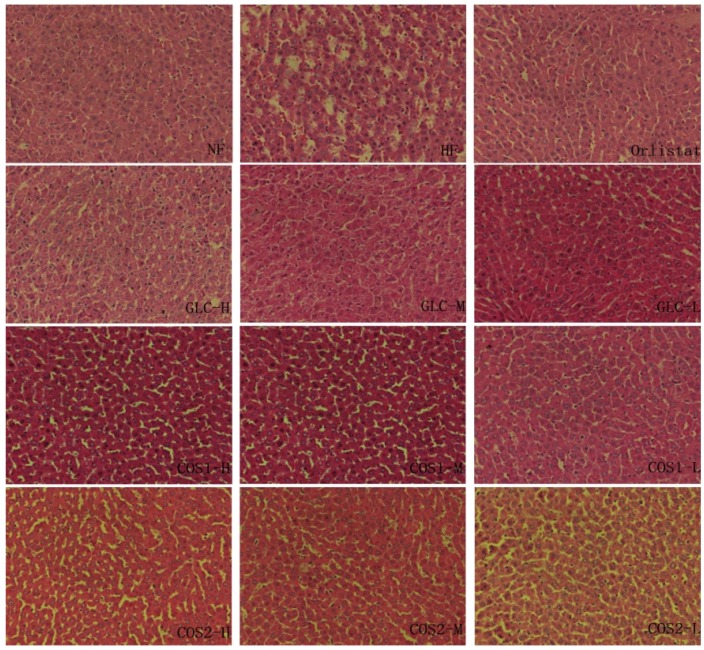
Histopathology of liver in rats. The liver tissues in the HF group developed a high degree of steatosis, hepatocytes with severe fat vacuoles and the infiltration of inflammatory cells. The symptoms of fatty liver were mitigated in varying degrees in Orlistat, GLC, COS1 and COS2 groups.

The pathology of the liver section was observed, and the results are presented in Figure 6. Histological abnormalities were not observed in the NF group. The liver histology showed fewer fat droplets in rats fed a normal diet. The rats in the HF group developed a high degree of steatosis, showing hepatocytes with severe fat vacuoles and the infiltration of inflammatory cells. The liver tissues of the Orlistat, GLC, COS1 and COS2 group in rats contained few fat vacuoles and few necrotic cells, similar to the NF group. The symptoms of liver steatosis were mitigated in the three GLC groups. Orlistat, GLC, COS1 and COS2 significantly alleviated signs of fatty liver compared to the HF group.

**Figure 7:**
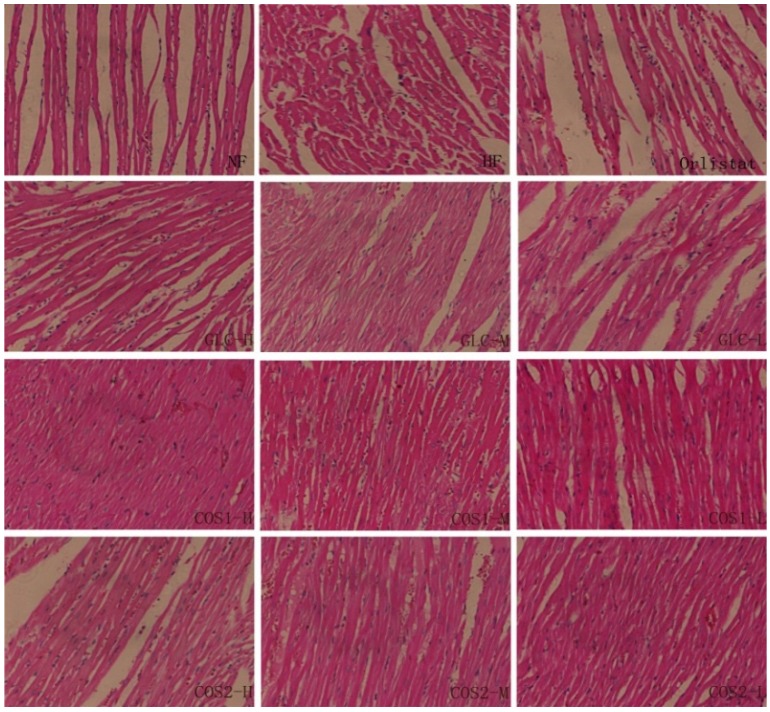
Histopathology of heart in rats. In the NF group, myocardial cells arranged orderly and cardiac muscle fibers showed clear. The heart sections of HF rats showed disarrayed myocardial fibers, interstitial fibrosis and the infiltration of inflammatory cells. Then, this state of rhabdomyolysis were alleviated in treatment groups.

The images of the heart pathology tissue sections are shown in Figure 7. The heart slices displayed no myocardial hypertrophy and the myocardial cells arrange orderliness which nucleus were normal without enlargement in the NF, Orlistat, GLC, COS1 and COS2 groups. The myofibers were ordered, and the muscle space and stripes were clear. The heart sections of HF rats showed disarrayed myocardial fibers, interstitial fibrosis and the infiltration of inflammatory cells.

**Figure 8:**
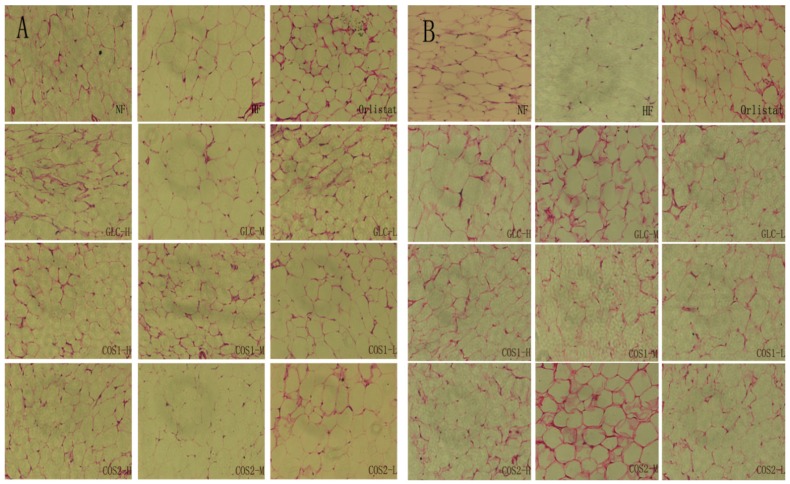
Histopathology of mesenteric fatty and subcutaneous fatty tissue. The adipocytes of the HF group were larger than those of other groups. Treatment with Orlistat, GLC, COS1 and COS2 clearly inhibited the proliferation of adipocytes. Note: (**A**) mesenteric fatty tissue; (**B**) subcutaneous fatty tissue.

The mesenteric fatty and subcutaneous fatty tissue morphology are presented in Figure 8. The adipocytes of the HF group were larger than those of other groups. Treatment with Orlistat, GLC, COS1 and COS2 clearly inhibited the proliferation of adipocytes. The groups treated with drugs showed fewer adipocytes cells than the HF groups for the same magnification and field of view.

### 2.7. Differential Gene Expression

#### 2.7.1. Test of RNA Quality

The results of the RNA quality test are shown in Table 4. The value of 28S/18S is closer to 2, which indicates a good RNA integrity that meets the requirements of constructing sequencing libraries. The integrity of each RNA sample (RIN) was satisfactory.

**Table 4:** The results of the RNA quality testing.

Sample	Instrument	RIN	Concentration (ng/μL)	28S/18S
NF	Agilent 2100	7.6	269	1.6
HF	Agilent 2100	7.8	298	1.5
GLC	Agilent 2100	7.2	300	1.6
COS1	Agilent 2100	7.8	310	1.5

#### 2.7.2. Differential Gene Expression Screening

**Figure 9:**
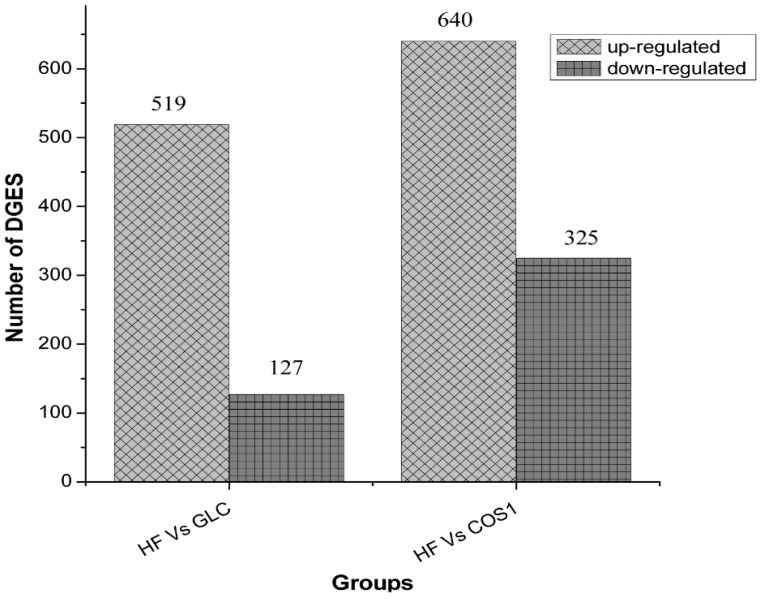
The Statistics of differentially expressed genes. Compared with the HF group, five hundred nineteen genes were up-regulated, while 127 were down-regulated in the GLC group. When COS1 group compared with the HF group, there were six hundred forty genes up-regulated and three hundred twenty five genes down-regulated.

The statistical results of the differential gene expression analysis are given in Figure 9. The differentially expressed genes are listed in Table 5 and Table 6. Six hundred forty-six genes were differentially expressed in the GLC group compared to the HF group. Five hundred nineteen genes were up-regulated, while 127 were down-regulated. The most up-regulated gene was Zfp354a (zinc finger protein 354a, Gene ID 24522), whose log2 Ratio (GLC/HF) was 9.07 (Gene | log2 Ratio (B/A) | ≥1, indicates a difference). The most down-regulated gene was Scd1 (stearoyl coenzyme A desaturase, Gene ID 246074), whose log2 Ratio (GLC/HF) was 8.36. Nine hundred sixty-five genes were differentially expressed in the COS1 group compared to the HF group. Six hundred forty genes were up-regulated, and 325 were down-regulated. The most up-regulated gene was RGD1311874 (hypothetical LOC300751, Gene ID 300751), whose log2 Ratio (COS1/HF) was 9.72. The most down-regulated gene was Ddx56 (DEAD (Asp-Glu-Ala-Asp) box helicase 56, Gene ID 289780), whose log2 Ratio (COS1/HF) was 10.00.

#### 2.7.3. GO Enrichment Analysis

The GO enrichment analysis showed that the differentially expressed genes in the GLC group compared to the HF group were mainly related to cell positions, such as organelle membrane, mitochondria, minute bodies and cytoplasm. Among others, the main molecular function was oxidoreductase activity, steroid dehydrogenation activity, NADH or NADPH as a donor, catalytic activity, ion binding, and purine nucleotide binding, among others. The enriched genes were mainly involved in biological processes that included metabolism, lipid metabolism, steroid metabolism, steroid metabolism, small molecule metabolites and fatty acid metabolism, among others.

The GO enrichment analysis showed that the differentially expressed genes in the COS1 group compared to the HF were mainly related to cell positions, such as organelles, ribosomes, mitochondria matrix and lysosomes. Among others, the main molecular function was catalysis, cofactor binding, steroid binding and hydrolysis activity, dehydrogenase activity, oxidoreductase activity and hydrolase activity. The enriched genes were mainly involved in biological processes that included metabolism, lipid metabolism, cell metabolism of ketones, organic acid metabolism, metabolic acid, steroid metabolism, metabolism of steroids and β-oxidation of fatty acids.

**Table 5:** The genes expressed of large differences (HF *vs.* GLC).

	Symbol	log2 Ratio (GLC/HF)	GO Function
Up-regulated	Zfp354a	9.07	GO:0001071//nucleic acid binding transcription factor activity;GO:0003676//nucleic acid binding;GO:0016564//transcription repressor activity;GO:0046914//transition metal ion binding;GO:0001071//nucleic acid binding transcription factor activity;GO:0003676//nucleic acid binding;GO:0016564//transcription repressor activity;GO:0046914//transition metal ion binding
	Fcrla	8.74	-
	Pbsn	7.91	GO:0005488//binding
	Clec4g	7.91	GO:0005488//binding
	Map2k6	7.85	GO:0004712//protein serine/threonine/tyrosine kinase activity; GO:0032559
Down-regulated	Scd1	8.36	GO:0016215;GO:0046914//transition metal ion binding;GO:0016215; GO:0046914//transition metal ion binding
	-	8.17	-
	Npas2	8.07	GO:0001071//nucleic acid binding transcription factor activity;GO:0003676//nucleic acid binding;GO:0031072//heat shock protein binding;GO:0060089
	Xcr1	7.13	GO:0001637//G-protein coupled chemoattractant receptor activity
	Cd99l2	7.02	-

#### 2.7.4. Pathway Significant Enrichment Analysis

In the GLC group, the genes were differentially expressed in 203 pathways. Table 7 lists the 11 significant enrichment pathways. The genes are generally significantly expressed in the pathway when the *Q* value ≤0.05. These 11 significantly enriched pathways were the metabolic pathway, circadian rhythm regulation, PPAR signaling pathway, the biosynthesis of primary bile acid, steroid hormones, steroid and backbone terpenoid, fatty acid metabolism, unsaturated fatty acids biosynthesis, lysosomes and bile secretion. Genes were differentially expressed in 210 pathways in the COS1 group. Table 8 lists the eight significant enrichment pathways, which are the metabolic pathway, biosynthesis of steroids, lysosomes, bile secretion, primary bile acid biosynthesis, retinol metabolism, circadian rhythm regulation and unsaturated fatty acid biosynthesis.

**Table 6:** The genes expressed of large differences (HF *vs.* COS1).

	Symbol	log2 Ratio (COS1/HF)	GO Function
Up-regulated	RGD1311874	9.72	-
	Zfp354a	9.25	GO:0001071//nucleic acid binding transcription factor activity;GO:0003676//nucleic acid binding;GO:0016564//transcription repressor activity;GO:0046914//transition metal ion binding;GO:0001071//nucleic acid binding transcription factor activity;GO:0003676//nucleic acid binding;GO:0016564//transcription repressor activity;GO:0046914//transition metal ion binding
	Pbsn	8.93	GO:0005488//binding
	Nkain3	8.52	-
	RGD1562667	8.36	GO:0005488//binding
Down-regulated	Ddx56	10	GO:0003676//nucleic acid binding;GO:0003724//RNA helicase activity;GO:0005515//protein binding;GO:0032559;GO:0042623//ATPase activity, coupled;GO:0003676//nucleic acid binding;GO:0003724//RNA helicase activity;GO:0005515//protein binding;GO:0032559;GO:0042623//ATPase activity, coupled
	LOC100362069	8.22	GO:0003676//nucleic acid binding;GO:0005198//structural molecule activity
	-	8.17	-
	-	7.64	GO:0004175//endopeptidase activity;GO:0005488//binding;GO:0004175//endopeptidase activity;GO:0005488//binding
	Paxip1	7.56	GO:0005488//binding;GO:0005488//binding

**Table 7:** The pathway of enrichment significantly (HF *vs.* GLC).

Pathway	DEGs with Pathway Annotation (502)	All Genes with Pathway Annotation (18,766)	*P* Value	*Q* Value	Pathway ID
Metabolic pathways	108 (21.51%)	2077 (11.07%)	5.821E−12	1.182E−09	ko01100
Circadian rhythm-mammal	8 (1.59%)	29 (0.15%)	6.489E−07	6.586E−05	ko04710
PPAR signaling pathway	16 (3.19%)	151 (0.8%)	3.001E−06	1.568E−04	ko03320
Primary bile acid biosynthesis	8 (1.59%)	35 (0.19%)	3.089E−06	1.568E−04	ko00120
Steroid hormone biosynthesis	10 (1.99%)	76 (0.4%)	3.366E−05	1.366E−03	ko00140
Steroid biosynthesis	6 (1.2%)	28 (0.15%)	8.117E−05	2.746E−03	ko00100
Terpenoid backbone biosynthesis	5 (1%)	25 (0.13%)	4.579E−04	1.328E−02	ko00900
Fatty acid metabolism	8 (1.59%)	70 (0.37%)	5.465E−04	1.387E−02	ko00071
Biosynthesis of unsaturated fatty acids	6 (1.2%)	43 (0.23%)	9.357E−04	2.111E−02	ko01040
Lysosome	13 (2.59%)	190 (1.01%)	1.861E−03	3.778E−02	ko04142
Bile secretion	10 (1.99%)	129 (0.69%)	2.469E−03	4.557E−02	ko04976

Note: DEGs with pathway annotation: the number of differentially expressed genes for the pathway; All genes with pathway annotation: the number of all genes noted to the pathway; *P* value obtained by hypergeometric test; *Q* ≤ 0.05, the pathway is enrichment pathway of differentially expressed genes; Pathway ID: ID number in KEGG database.

**Table 8:** The pathway of enrichment significantly (HF *vs.* COS1).

Pathway	DEGs with Pathway Annotation (744)	All Genes with Pathway Annotation (18,766)	*P* Value	*Q* Value	Pathway ID
Metabolic pathways	135 (18.15%)	2077 (11.07%)	3.405E−09	5.525E−07	ko01100
Steroid hormone biosynthesis	17 (2.28%)	76 (0.4%)	5.261E−09	5.525E−07	ko00140
Lysosome	23 (3.09%)	190 (1.01%)	1.968E−06	1.377E−04	ko04142
Bile secretion	18 (2.42%)	129 (0.69%)	3.394E−06	1.782E−04	ko04976
Primary bile acid biosynthesis	9 (1.21%)	35 (0.19%)	6.438E−06	2.704E−04	ko00120
Retinol metabolism	11 (1.48%)	86 (0.46%)	5.761E−04	2.016E−02	ko00830
Circadian rhythm-mammal	6 (0.81%)	29 (0.15%)	8.272E−04	2.482E−02	ko04710
Biosynthesis of unsaturated fatty acids	7 (0.94%)	43 (0.23%)	1.388E−03	3.643E−02	ko01040

Note: DEGs with pathway annotation: the number of differentially expressed genes for the pathway; All genes with pathway annotation: the number of all genes noted to the pathway; *P* value obtained by hypergeometric test; *Q* ≤ 0.05, the pathway is enrichment Pathway of differentially expressed genes; Pathway ID: ID number in KEGG database.

#### 2.7.5. PPAR Signaling Pathway

**Figure 10:**
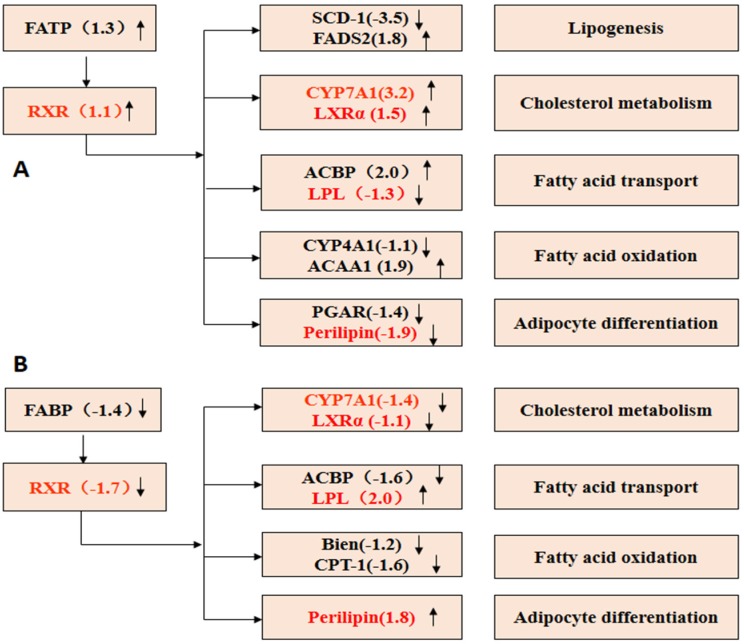
PPAR signaling pathway. (**A**) HF *vs.* NF PPAR Signaling Pathway; (**B**) COS1 *vs.* NF PPAR Signaling Pathway. **↑** Gene was up-regulated; **↓** Gene was down-regulated.

Compared with the NF group, retinoid receptor X (RXR), cholesterol 7α-hydroxylase (CYP7A1) and acetyl coenzyme A binding protein (ACBP), which play important roles in the conversion of cholesterol and fat transport, were up-regulated in the HF group (Figure 10A). Equally, there were some genes differentially expressed in the COS1 group compared to the NF group (Figure 10B), and four of these genes expressed were different from those in the HF group. CYP7A1 and liver X receptor (LXRα) were down-regulated, perilipin and lipoprotein lipase (LPL) were up-regulated in the COS1 group. Conversely, CYP7A1 and LXRα were up-regulated, while perilipin and LPL were down-regulated in the HF group.

CYP7A1 is a lipid metabolism gene regulated by PPARα, which participates in the PPAR signaling pathway. Activated PPARα forms a complex with RXR and combines with CYP7A1 regulatory elements (PPRE) to regulate the transcription of CYP7A1. Pharmacodynamic experiments showed that GLC markedly lowered cholesterol to likely significantly reduce serum CHO by up-regulating RXR, which increased the expression of the CYP7A1 gene and thus accelerated the conversion of cholesterol into bile acids. The up-regulated expression of ACBP, increase in the combination rate with the membrane or intracellular fatty acids and their derivatives to form protein complexes, or reduction in fat synthesis may regulate the conversion of fatty acids. In summary, GLC may be a potential drug to lower lipid levels and aid weight loss.

LXRα binds to RXR to form the LXRα-RXRα complex in the liver. The complex combines with binding site 1 to the activate transcription of CYP7A1. Therefore, CYP7A1 and LXRα in the PPAR signaling pathway were up-regulated in the COS1 group, which regulates the metabolism of cholesterol. LPL helps TG, VLDL and chylomicrons to convert into glycerol and FFA in the serum. The down-regulation of LPL in the COS1 group added to the lipid transport of VLDL and chylomicrons, which could be broken into fatty acids in muscle tissue. Perilipin inhibits the decomposition of TG and increases lipid storage during feeding. The down-regulation of perilipin in the COS1 group is reflected by the inhibition of lipid storage. In summary, COS1 could be a potential drug to lower lipid levels and aid weight loss.

### 2.8. Expression of PPARγ and LXRα mRNA in Epididymal Adipose Tissue

**Figure 11:**
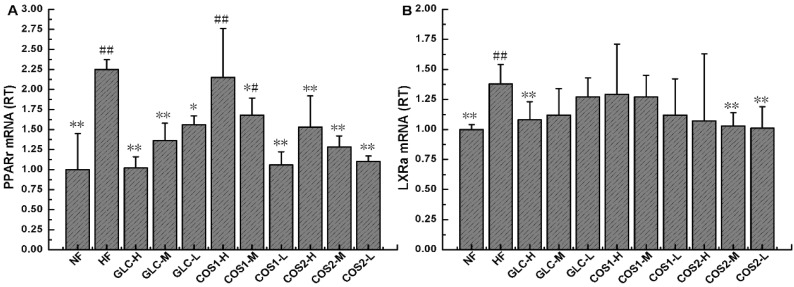
The mRNA expression levels of epididymal fat PPARγ (**A**) and LXRα (**B**) in the different groups. The level of mRNA was detected by Q-PCR. ΔCt is the average value of 10 samples in the formulation (average mRNA expression of experiment groups/average mRNA expression of NF) = 2^−∆∆Ct^ = 2^−(∆Ct control − ∆Ct FF)^. If 2^−∆∆Ct^ < 1, the average mRNA expression of the experiment groups is lower than that in NF. If 2^−∆∆Ct^ > 1, the average mRNA expression of the experiment groups is higher than that in NF. Note: Compared with HF, * *P* < 0.05; ** *P* < 0.01; Compared with NF, ^#^ *P* < 0.05; ^##^ *P*< 0.01.

To explore the pharmacological mechanisms of GLC, COS1 and COS2, the expressions of PPARγ and LXRα mRNA were measured in rat epididymal adipose tissues using real-time quantitative PCR. Figure 11A demonstrated that the mRNA expression of PPARγ decreased in response to different doses of GLC, COS1 and COS2, and these differences were statistically significant compared to the HF group (*P* < 0.05). Notably, the effect was the most pronounced in the GLC-H, COS1-L and COS2-L groups. As displayed in Figure 11B, GLC, COS1 and COS2 effectively reduced the LXRα mRNA levels in the epididymal adipose tissues of rats fed a high fat diet when compared with the HF group (*P* < 0.05).

### 2.9. Discussion

The present study was carried out to investigate the anti-obese and hypoglycemic activities of GLC, COS1 and COS2 in obese rats. The results indicated that GLC could reduce the weight and body fat of obese rats while minimally decreasing appetite, which demonstrated its anti-obese effect to some extent. Moreover, GLC could significantly reduce the levels of CHO and LDL-C in the serum and attenuate liver cell necrosis, which effectively lowered the blood lipid level and protected the liver; these effects were more significant than those observed for Orlistat. COS1 and COS2 also significantly inhibited weight increase in rats, and liver fat deposition was reduced in the following order: COS1 > Orlistat > COS2.

Some studies have shown that low-molecular weight chitosan is a more effective anti-obese and anti-diabetic agent in high-fat diet induced obesity and diabetic animal models, respectively [35,36]. COS1 significantly reduced the CHO and LDL-C levels in the serum, followed by orlistat and COS2. The deposition of hepatic lipids was reduced, and liver cell necrosis was improved in the COS1 and COS2 groups, suggesting that these drugs somewhat reduce the lipid levels.

The perirenal adipose tissue and epididymal adipose tissue samples were taken to calculate the wet weight of the fat pad and body fat ratio. The wet weight of the fat pad in HF was higher than that in the NF group, indicating that the fat content of the HF group was increased. Compared with the HF group, Orlistat, GLC-H, and COS2-H (*P* < 0.05) could reduce body fat and the body fat ratio in obese rats. COS1 could also reduce body fat in rats, but this difference was not significant. The DGE analysis showed that 646 genes were differentially expressed in the GLC group compared to the HF group, including 519 up-regulated genes and 127 down-regulated genes. Nine hundred sixty-five genes were differentially expressed in the COS1 group compared to the HF group, including 640 up-regulated genes and 325 down-regulated genes. The GO enrichment analysis showed that the enriched genes were mainly involved in biological processes, including lipid, cell ketones, organic acid and steroid metabolism as well as the β-oxidation of fatty acids. The differentially expressed genes distributed in 203 pathways in the GLC group and in 210 pathways in the COS1 group. Some of these significantly enriched pathways were metabolic in nature and included the biosynthesis of steroids, lysosomes, bile secretion, primary bile acid biosynthesis, retinol metabolism, circadian rhythm regulation and unsaturated fatty acid biosynthesis. An analysis of the PPAR signaling pathway indicated that the retinoid receptor X (RXR) was up-regulated in the GLC group. RXR plays an important role in the conversion of cholesterol and fat transport. PPARs can form a complex with RXR to regulate lipid metabolism after activation. We also found that the LXRα/PPARs signaling pathway, which regulates the differentiation of adipocytes, was up-regulated in the COS1 group. The peroxisome proliferator-activated receptors (PPARs), which respond to fatty acids [37], and liver X receptors (LXRs), which respond to oxysterols and glucose [38,39], are a family of ligand-activated transcription factors called nuclear receptors and have emerged as crucial regulators of cellular metabolism [40]. The PPAR family includes PPARα, PPARβ, and PPARγ. The prevalence of these receptor subtypes varies by tissue, and PPARγ is the most prevalent subtype in adipose tissue [41]. In clinical practice, PPARγ agonists (thiazolidinediones) are used to increase insulin sensitivity in adipose tissue and muscle [42,43], whereas PPARα agonists (fibrates) are used to treat hyperlipidemia. PPARγ controls both pre-adipocyte differentiation and lipid storage [44] and is recognized as a key regulator of adipocyte function. The LXR family includes both LXRα and LXRβ [38,39,45]. The latter is ubiquitously expressed, while the former is most highly expressed in the liver, adipose tissue, and macrophages [46,47] and is activated by glucose and sterols. In the liver, LXRα activates lipogenic and glycolytic genes following activation by its ligands partly via the activation of sterol regulatory element binding proteins (SREBPs) [48,49]. LXRs activate the coordinated expression of major fatty acid biosynthetic genes (lipogenesis) and increase plasma triglyceride and phospholipid levels in rats [50]. Following a ligand binding event, both PPARs and LXRs become activated and heterodimerize with RXR [40].

**Figure 12:**
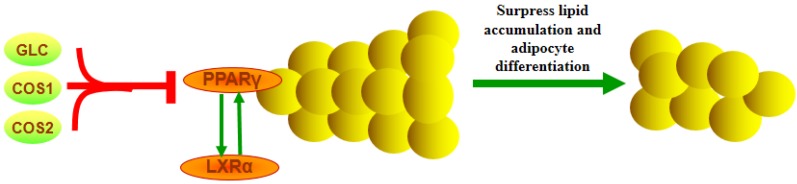
PPARγ and LXRα mRNA in adipose tissue. PPARγ and LXRα function as obligate heterodimers with retinoid X receptors (RXR) and influence gene transcription via multiple mechanisms. In keeping with their functions as lipid sensors, ligand-bound PPAR or LXR activate feed-forward metabolic cascades that regulate lipid homeostasis via the transcription of genes involved in lipid metabolism, storage, and transport. PPARγ activates a battery of genes involved in lipid storage and lipogenesis, allowing adipose tissue safely to store greater quantities of fat and undergo differentiation.

To explore the pharmacological mechanisms of GLC, COS1 and COS2, we measured the relative transcript levels (RT) of PPARγ and LXRα mRNA in the epididymal adipose tissue of rats using real-time quantitative PCR. Our results showed that the differentiation of adipocytes stimulated the expression of PPARγ mRNA [31]. As demonstrated in Figure 11A, treatment with GLC resulted in a desired dose-dependent inhibition of PPARγ mRNA expression (*P* < 0.01). The HF group expressed more PPARγ mRNA than the NF group. Treatment with COS1 and COS2 also significantly reduced the PPARγ mRNA expression compared to the HF group (*P* < 0.05). As shown in Figure 11B, GLC, COS1 and COS2 suppressed the expression of LXRα compared to the HF group, suggesting that these drugs may regulate adipogenesis via the modulation of PPARγ expression (Figure 12). The combined effects on peripheral metabolism and lipid homeostasis have made LXRα and PPARγ major targets in the treatment of obesity, diabetes, and cardiovascular disease.

## 3. Materials and Methods

### 3.1. Materials

COS1 and COS2, with a number-average molecular weight of ≤1000 and ≤3000, respectively, were purchased from Laizhou Haili Biological Products Co. Ltd., Shandong, China. GLC was in hydrochloride form and was also purchased from Laizhou Haili Biological Products Co. Ltd., Shandong, China. Orlistat capsules were obtained from Chongqing Fortune Pharmaceutical Co. Ltd., Chongqing, China. Total cholesterol (CHO), triacylglycerol (TG), high-density lipoprotein cholesterol (HDL-C) and low-density lipoprotein cholesterol (LDL-C) kits were obtained from BioSino Biotechnology and Science Inc., Beijing, China. All other reagents and solvents were of analytical grade and directly used without further treatment.

### 3.2. Animals

All experiments and animal protocols were approved by the Institutional Animal Care and Use Committee of Guangdong Pharmaceutical University (Guangzhou, China) (Protocol no. SPF2013100). A total number of 185 male Sprague-Dawley rats aged 4 weeks were used in these experiments and purchased from Guangdong Medical Laboratory Animal Center (Guangzhou, China). The rats were housed in cages under standard conditions: 22 ± 1 °C, a 12-h light and dark cycle, and a relative humidity of 50%~60%. The rats were allowed to acclimatize for 1 week prior to the experiments.

### 3.3. Experimental Design

The 185 rats were divided into two groups after the one-week adaptation period. Twenty rats were fed with a normal diet (standard rodent chow) and served as the control group (NF), and the remaining rats were fed with a high-fat diet. This high-fat diet consisted 54% basic feed, 15% lard, 15% sucrose, 4% milk powder, 3% peanut, 5% egg yolk powder, 1% sesame oil, 2% salt, 0.6% dicalcium phosphate and 0.4% mountain flour. After two weeks of feeding, the rats fed with a high-fat diet were sorted by weight gain, and the obesity-resistant rats that gained less weight were eliminated. The obesity-sensitive rats were selected and fed with a high-fat diet for another 6 weeks. The obesity model was considered to be complete when the average weight of the rats fed with a high-fat diet exceeded 20% of the weight of rats fed with a normal diet.

The 110 obesity-sensitive rats were then randomly divided into 11 groups: high-fat diet group (HF), Orlistat control group (Orlistat), GLC high, middle, low dose group (GLC-H, GLC-M, GLC-L), COS1 high, middle, low dose group (COS1-H, COS1-M, COS1-L), COS2 high, middle, low dose group (COS2-H, COS2-M, COS2-L). The NF group constituted the 12th group. The test samples were administered orally by gavage at a dose of 1 mL/100 g per day at the same time for 5 weeks. The rats in the HF and NF groups were given an equal volume of distilled water. The test samples were dissolved in distilled water. The doses of test samples for each group were as follows: Orlistat, 75 mg/kg/day; GLC-H, 600 mg/kg/day; GLC-M, 300 mg/kg/day; GLC-L, 150 mg/kg/day; COS1-H, 1000 mg/kg/day; COS1-M, 500 mg/kg/day; COS1-L, 250 mg/kg/day; COS2-H, 1000 mg/kg/day; COS2-M, 500 mg/kg/day; and COS2-L, 250 mg/kg/day. During the administration, all rats were weighed weekly, and the food intake levels for each group were recorded daily. The rats had free access to food and water. The rats were fasted for 16 h and dissected. The blood was collected from the abdominal aorta, and their perirenal fat and epididymal fat were withdrawn to calculate the wet weight of the fat pad and body fat ratio (wet weight of fat pad/body weight). The liver and heart were collected to calculate the organ index (wet weight of the organs/body weight) for the experiments described below.

### 3.4. Biochemical Parameter Analysis

At the end of the treatment period, the rats were fasted overnight, and their blood samples were taken from abdominal aorta after receiving 1% sodium pentobarbital (0.5 mL/100 g BW) anesthesia. The serum samples were prepared by centrifuging the collected blood samples (3500 r·min^−1^ for 15 min), and the samples were then stored at −80 °C prior to the biochemical tests. The total cholesterol (CHO), triacylglycerol (TG), high-density lipoprotein cholesterol (HDL-C), and low-density lipoprotein cholesterol (LDL-C) serum concentrations were measured with commercial assay kits using an automated biochemistry analyzer BC200 instrument (BC200, Beijing Precil Instrument Co. Ltd., Beijing, China).

### 3.5. Histological Analysis

The liver, heart, mesenteric fat and subcutaneous fat tissues were cut into 0.5-cm^3^ pieces, washed with saline, and placed in the tissue cassette. The cassettes were marked with a pencil and then placed into 12% formaldehyde solution for 24 h to fix the tissue. The residual fixative was washed away with distilled water. These tissues were dehydrated using 30%, 50%, 70%, 80%, 90%, 95% and 100% ethanol, embedded in paraffin (BMJ-III embedding machine, Changzhou Electronic Instrument Factory, Jiangsu, China), and then cut into 5-μm thick sections using a microtome (Leica RM2235; Leica, Heidelberg, Germany). The tissues were stained with hematoxylin and eosin (H&E) and observed under a microscope at 200× magnification.

### 3.6. Digital Gene Expression Tag Profiling

The liver RNA of the NF, HF, GLC and COS1 groups was extracted with Agilent RNA 6000 pico kits (Agilent Technologies, Santa Clara, CA, USA). The concentration and quality was detected using an Agilent 2100 instrument (Agilent Technologies, Santa Clara, CA, USA). Beijing Genomics Institution (BGI) conducted the DGE.

### 3.7. Quantitative RT-PCR Analysis

The rats were sacrificed, and the epididymal fat was collected and stored at −80 °C. The total RNA was isolated from these issues using TRIzol reagent (Invitrogen, Inc., Carlsbad, CA, USA). Single-stranded cDNA was synthesized from 1 µg of total RNA using the PrimeScript TM RT reagent kit with gDNA Eraser (TaKaRa, Code NO. RR047A, Shiga, Otsu, Japan) at the following conditions: 37 °C for 15 min, 85 °C for 5 s and storage at 4 °C. The cDNA products were quantified by real-time RT-PCR using a TaKaRa SYBR Premix Ex Taq™ kit (TaKaRa, Code NO. RR420A, Shiga, Otsu, Japan) and the Bio-Rad IQ5 real-time PCR system and analysis software (Applied Biosystems, Carlsbad, CA, USA). The primer sequences used for PCR were designed and synthesized by Sangon Biotech Co. Ltd. (Shanghai, China). Beta-actin (β-actin) was used as the internal control (Housekeeping gene). The sequences of primers used for amplification were defined as follows:

PPARγ (Sequence ID: NM_001145366.1)
  FORWARD: 5′-GCCCTTTGGTGACTTTATGG-3′
  REVERSE: 5′-CAGCAGGTTGTCTTGGATGT-3′
LXRα (Sequence ID: NM_031627.2)
  FORWARD: 5′-GCACGCTACATTTGCCATAG-3′
  REVERSE: 5′-CCTGCTCCTCTTCTTGACG-3′
β-actin (Sequence ID: NM_031144)
  FORWARD: 5′-CACCCGCGAGTACAACCTTC-3′
  REVERSE: 5′-CCCATACCCACCATCACACC-3′

These primers were used for the quantitative RT-PCR, and PCR was performed according to the following protocol: 95 °C for 30 s (initial denaturation), followed by 39–40 cycles at 95 °C for 5 s and 60 °C for 30 s. Melt curve analyses were performed with each series to confirm the specificity of the primers and amplified products using the following parameters: the amplified products were heated from 65 °C to 95 °C at 0.5 °C steps for 5 s. The relative quantification of mRNA expression was analyzed using the 2^−∆∆Ct^ method.

### 3.8. Data Analysis

All data are presented as the means ± standard deviation (SD). The effects of different treatments were compared with a one-way ANOVA test using SPSS for Windows, version 8.0 (OriginLab, Northampton, MA, USA). *P* < 0.05 was considered statistically significant.

## 4. Conclusions

In summary, the anti-obese effects of GLC, COS1 and COS2 show that these compounds are potentially important dietary supplements for the prevention and attenuation of obesity and related disorders. However, further studies to illuminate the mechanisms of action and their relative influence on biological functions, including human studies, are necessary.

## Abbreviations

COS: chitosan oligosaccharide
GLC: glucosamine
CTS: chitosan
NF: normal diet group
HF: high-fat diet group
CHO: total cholesterol
TG: triacylglycerol
HDL-C: high-density lipoprotein cholesterol
LDL-C: low-density lipoprotein cholesterol
PPAR: peroxisome proliferator-activated receptor
LXR: liver X receptor
